# Immunologists’ perspective of nephropathology

**DOI:** 10.15171/jnp.2016.11

**Published:** 2016-03-18

**Authors:** Sabiha Anis

**Affiliations:** ^1^Department of Immunology and Molecular Biology, Sindh Institute of Urology and Transplantation, Karachi, Pakistan

**Keywords:** Immunopathogenesis, Inflammation, Nephropathology, Renal disorders

**Implication for health policy/practice/research/medical education:**

An awareness and complete knowledge of the immunopathogenesis of renal disorders is mandatory. This is important not only for classifying disease, but in fact an effective management can only be planned according to the etiopathogenesis involved in the disease which is essential for the benefit of the patient.

Majority of renal diseases, especially those involving the glomeruli and blood vessels, are caused by immune mediated injury. Either systemic or local abnormal immune responses are known to cause renal pathology. The abnormality may be due to hyperactive or a deficient immune system (1,2). The abnormal immune mechanism is not limited to the etiopathogenesis of glomerulonephritis (GN), but recurrent and/or severe urinary tract infections (UTI) may also occur due to immune aberrancy (1,3). Both innate and adaptive immune systems are involved in the pathogenesis of renal disorders (1,4). In this editorial, the cells and molecules of both innate and adaptive immune system involved in the pathogenesis of renal diseases will be discussed briefly.

Our immune system is broadly divided into innate and adaptive immune systems. The cells of the innate immunity are polymorph nuclear leucocytes, monocytes/macrophages, natural killer cells and dendritic cells. Numerous molecules are involved in the innate immune response network such as complement proteins, C-reactive proteins and other acute phase proteins, receptors for pathogen associated molecular patterns (PAMPs), enzymes and cytokines. The adaptive immune system mainly includes T and B lymphocytes, antibodies, cytokines and numerous transcription factors involved in differentiation and generation of specific immune response to damaging agents. The innate and adaptive immune responses are very well coordinated and tightly regulated. It is when this coordination and regulation fails, that diseases occur (2,3,5,6).

The urinary tract system bears the main burden of removing waste and maintaining homeostasis in the body (1,6). Various renal disorders cause abnormalities of both innate and adaptive immune system in turn leading to increased incidence of infections and inflammatory disorders of the system (7). Similarly the immune system has a major role in maintaining physiology and causing pathology of urinary tract system (8). Knowledge of coordination of these two systems is important not only to understand the pathogenesis but also helps in the complete management of the patient including diagnostic workup, treatment and monitoring.

The cells and the molecules of innate immune system have a major role in maintaining immune surveillance, providing protection to the urinary tract system against pathogens and other damaging factors (9). The immune response is initiated with the recognition of PAMPs and damage associated molecular patterns (DAMPs). Several pathogen recognition receptors (PRRs) are present on our immune cells as well as glomerular and tubular cells that recognize these molecular patterns. The importance of physiology and pathology of PRRs in urinary tract system and renal disorders are now being increasingly recognized. The most important and very well studied are toll like receptors (TLRs). They not only initiate innate immune response, but also stimulate specific immunity against the inciting agents. They are also responsible for the stimulation of secretion of many inflammatory cytokines and chemokines, leading to tissue injury (2,10).

The release of cytokines and chemokines upon ligation of PRRs leads to the recruitment of cells including phagocytes and lymphocytes to the area of injury. The neutrophils and monocytes cause injury due to the generation of reactive oxygen species upon excessive activation. The lymphocytes also participate in the damage by augmenting the immune response to cognate antigens (11,12).

The inflammasomes causing inflammation have a very important role in renal injury. NLRP3 inflammasomes can be stimulated by bacterial products, uric acids or apoptotic cell debris directly or indirectly by DAMPs. This, in turn, activates IL-1 converting enzyme leading to increased expression of IL-1 and inflammation (13). This is enhanced in the presence of genetic defects leading to excessive inflammation and autoimmunity. NLRP3 is also probably linked to renal scarring and chronic kidney disease (1,10).

Complement activation is important not only for the elimination of uropathic pathogens but also for the removal of injurious immune complexes that cause damage to urinary tract system. The alternate and lectin pathways are the components of innate immune response armamentarium, while the classical pathway is activated by antigen-antibody complexes. The inefficient activation or ineffective control of this system is not only associated with severe UTI but also leads to renal injury due to excessive and uncontrolled inflammation. Excessive and/ or aberrant activation of any or all three complement pathways is seen in various GNs (1,14). The mutations or deficiencies of complement regulatory proteins are associated with severe renal damage secondary to excessive alternate pathway activation (15).

The important role of antibodies and mediation of inflammation is very well known in causing GN. These may be autoantibodies against various local or systemically generated antigens. They can cause pathogenesis by directly binding to native or to localized antigens, or may get deposited in the form of antigen antibody complexes in various renal tissues. The ultimate activation of complement system, recruitment of inflammatory cells, surge of chemokines and cytokines all lead to renal injury (2).

Hence, depending on the etiology and pathogenesis, the treatment strategies are better planned. These may include steroidal and non-steroidal anti-inflammatory drugs, anti-hypertensives, immunosuppressive drugs, biomolecules including monoclonal antibodies, plasmapheresis and others (6,16,17).

## Author’s contribution

SA was the single author of this manuscript.

## Conflicts of interest

The authors declared no competing interests.

## Funding/Support

None.
